# Supplementary material for: A COVID-19 DNA Vaccine Candidate Elicits Broadly Neutralizing Antibodies against Multiple SARS-CoV-2 Variants including the Currently Circulating Omicron BA.5, BF.7, BQ.1 and XBB
Source: Vaccines (Basel). 2023 Mar 31;11(4):778. doi: 10.3390/vaccines11040778 (PMC10144402; doi:10.3390/vaccines11040778)
Supplement: Supplementary file 1 [file vaccines-11-00778-s001.zip › vaccines-2238015-supplementary.pdf]

A: Cloning strategy of pAD1016

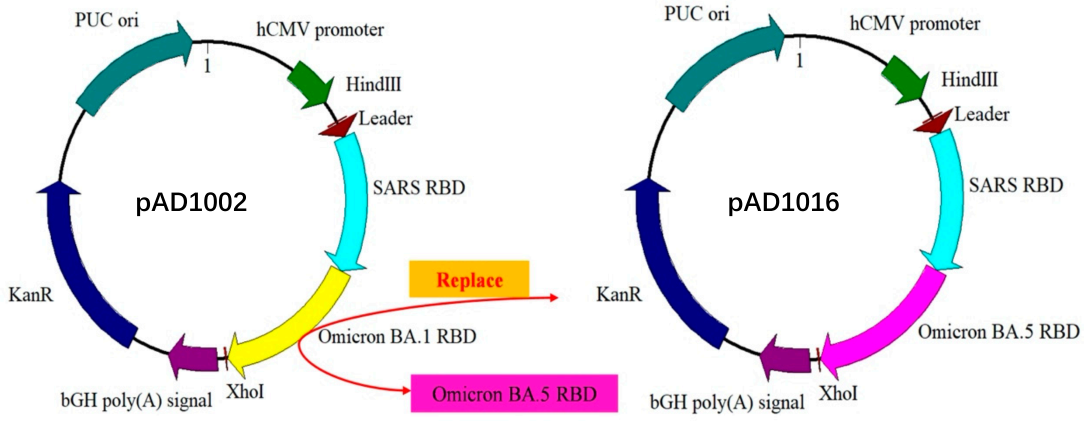

B: Amino acid sequence of antigen encoded by pAD1016

MWWRLWWLLLLLLLLWPMVWANITNLCPFGEVFNATKFPSVYAWERKKISNCVADYSVLYN
STFFSTFKCYGVSATKLNDLCFSNVYADSFVVKGDDVRQIAPGQTGVIADYNYKLPDDFMG
CVLAWNTRNIDATSTGNYNYKYRYLRHGKLRPFERDISNVPFSPDGKPCTPPALNCYWPLN
DYGFYTTTGIGYQPYRVVVLSFELLNAPATVCGPRVQPTESIVRFPNITNLCPFDEVFNATRF
ASVYAWNRKRISNCVADYSVLYNFAPFFAFKCYGVSPTKLNDLCFTNVYADSFVIRGNEVS
QIAPGQTGNIADYNYKLPDDFTGCVIAWNSNKLDSKVGGNYNYRYRLFRKSNLKPFERDIS
TEIYQAGNKPCNGVAGVNCYFPLQSYGFRPTYGVGHQPYRVVVLSFELLHAPATVCGPKKS
TNLVKNK

Signal Peptide — SARS RBD—Omicron BA.5 RBD (no linker between RBDs).

Supplemental Figure S1. Clonal strategy and structural characteristics of plasmid pAD1016. (**A**) Schematic diagram showing the cloning strategy and structural characteristics of vaccine candidate pAD1016 encoding heterodimeric fusion RBDs of SARS-CoV-1 and SARS-CoV-2 Omicron BA.5 with a secretion leader sequence. (**B**) Complete amino acid sequence of the antigen encoded by pAD1016 is shown.

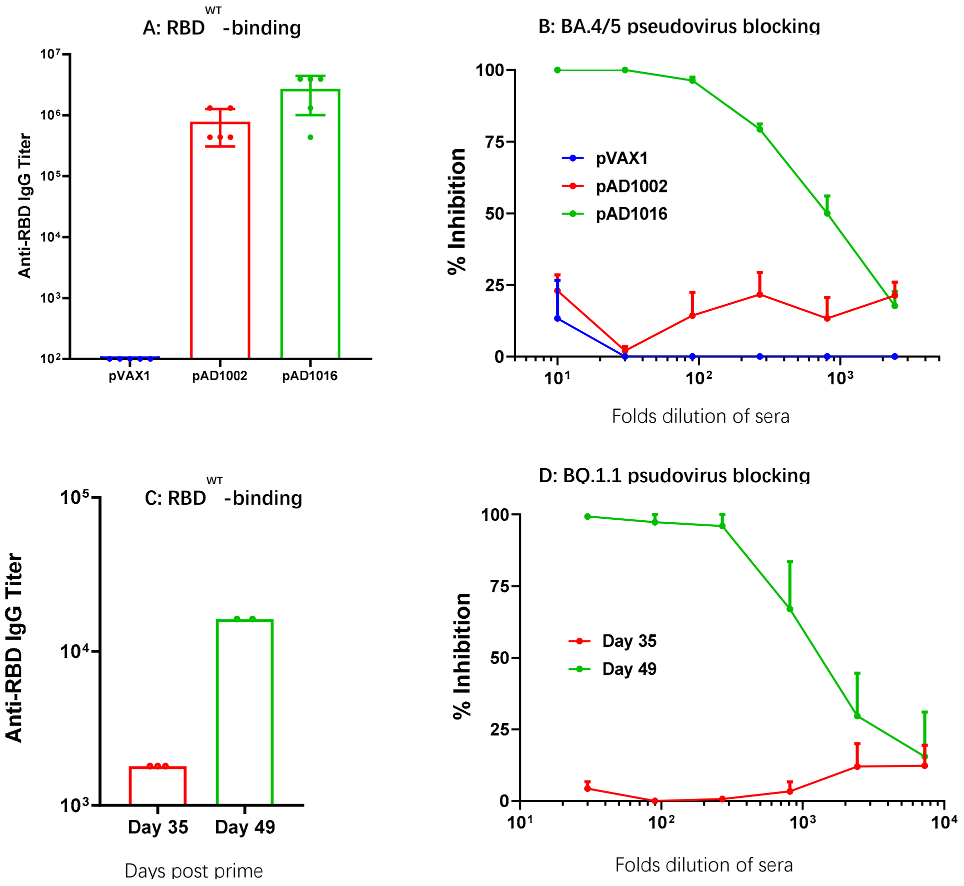

Supplemental Figure S2. pAD1016-induced binding and neutralizing Abs in hACE2-transgenic K18 mice and pigs. hACE2-transgenic K18 mice (n=5) were IM+EP immunized twice with either pVAX1, or pAD1002, or pAD1016 (20 µg/dose, fortnight interval). Serum samples, collected 14 days post boost, were tested for ability to bind recombinant RBD^{WT} in ELISAs (**A**) and to block mimic infection of ACE2-expressing HEK293T cells by pseudo-viruses displaying S protein of SARS-CoV-2 BA.4/5 (**B**). Female piglets (n=3) were administered with 500µg pAD1016/IM+EP on Days 0 and 35. Serum samples from the immunized pigs, collected on Days 35 and 49 post primary immunization, were tested for ability to

bind recombinant RBD^{WT} in ELISAs (**C**) and to block mimic infection of ACE2-expressing HEK293T cells by pseudoviruses displaying S protein of Omicron BQ.1.1 (**D**). The results are expressed as endpoint dilution titers for RBD-binding serological IgG in ELISAs (**A, C**) and percent inhibition of infection in pseudovirus neutralization assays (**B, D**). Data are means ± SEM.

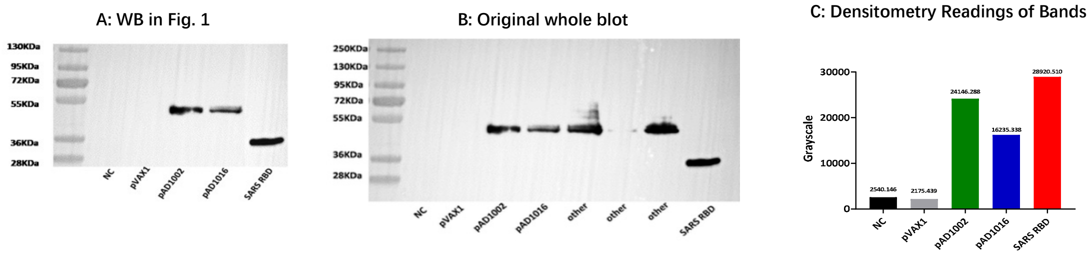

Supplemental Figure S3. Original whole blot and the densitometry readings of the bands in WB shown in Fig. 1.
